# “I Just Don’t Know What to Believe”: Sensemaking during the COVID-19 Pandemic among Criminal Legal Involved Communities

**DOI:** 10.3390/ijerph192215045

**Published:** 2022-11-15

**Authors:** Rochelle Davidson Mhonde, Breonna Riddick, Aayushi Hingle, Cameron Shaw, Danielle Rudes, Harold Pollack, John Schneider, Xiaoquan Zhao, Faye S. Taxman

**Affiliations:** 1 Department of Global and Community Health, George Mason University, Fairfax, VA 22030, USA; 2 Department of Communication, George Mason University, Fairfax, VA 22030, USA; 3 Schar School of Policy and Government, Center for Advancing Correctional Excellence, George Mason University, Fairfax, VA 22030, USA; 4 College of Criminal Justice Huntsville, Sam Houston State University, Huntsville, TX 77341, USA; 5 Crown Family School of Social Work, Policy and Practice, University of Chicago, Chicago, IL 60637, USA; 6 Departments of Medicine and Public Health Sciences, University of Chicago, Chicago, IL 60637, USA

**Keywords:** criminal legal involvement, COVID-19, misinformation, public health, race, trust, vulnerable populations

## Abstract

During the COVID-19 pandemic in the United States, those involved with the criminal legal system experience disproportionate vulnerability to infection, transmission, and mortality, facing additional systemic barriers due to criminal legal involvement (CLI) (e.g., prior incarcerations or probationary status affecting employability or housing security). We use Weick’s (1979) model of sensemaking as a theoretical framework to inform our examination of CLI individuals’ experiences during the pandemic. The primary objective of this paper is to explore the process of sensemaking amid misinformation, trust/mistrust, and vulnerability during the pandemic among CLI communities in three central states (Illinois, Louisiana, and Arkansas). We conducted seven online focus groups (*n* = 44), between December 2020 and January 2021, from the targeted communities about their awareness of misinformation, trusted or distrusted sources, attitudes about COVID-19 health behaviors (including testing, protective behaviors such as mask-wearing and social distancing, and vaccination), and experiences with the criminal legal system during the pandemic. The concept of equivocality was at the core of the narratives shared among participants, with *uncertainty* emerging as a meta-theme across all focus groups. The findings of this study should prove useful for those who are developing messaging to combat mis/disinformation and overcome mis/distrust with the medical system and government institutions among those who are disenfranchised.

## 1. Introduction

Marginalized and minoritized communities in the United States face exacerbated health disparities during the COVID-19 pandemic. Individuals identifying as Black, Indigenous, Hispanic, Latinx, or Pacific Islander experience higher COVID-19 hospitalizations and death rates than other racial groups [1,2,3,4,5]. These communities are more likely to face economic and structural disparities because of systemic racism, unequal access to healthcare, and higher rates of comorbidities that increase the risk of infection and mortality [3,6,7]. Those involved with the criminal legal system experience even greater vulnerability to infection, transmission, and mortality [8,9], facing additional systemic barriers due to criminal legal involvement (e.g., prior incarcerations or probationary status affecting employability or housing security). As the pandemic continues, vulnerabilities related to employment, housing safety, food security, health, and well-being have been magnified [9,10,11]. Thus, it is critical to examine intensified risks of COVID-19 for minoritized communities, criminal legal involved (CLI) individuals, and the intersection of these two groups.

At the end of 2020, about 5,500,600 people were involved in the US criminal legal system, including 1,691,600 incarcerated in federal prison or local jail and 3,890,400 on probation or under parole supervision [12]. Racial and ethnic minoritized groups continue to be disproportionately represented, with Black Americans incarcerated at five times the rate and Latinx populations at 1.3 times the rate of White Americans [13]. In 2021, Black and Latinx people comprised 30 percent of the US population but accounted for over half of the incarcerated population [12,13]. The vast majority of people impacted by the criminal legal system come from minoritized communities, which calls for further examination into the disproportionate adverse health outcomes faced by those at the intersections of racial and CLI identities.

Among the health disparities faced by individuals involved in the criminal legal system, those in federal or state prisons are 1.5 times more likely to report having a chronic condition such as asthma, heart disease, or diabetes; have a higher rate of transmission of infectious disease; and are more likely to experience mental health issues, psychological distress, and substance abuse disorders when compared to the general US population [14]. These health disparities can be partially attributed to racial and ethnic predispositions to illness, calling for research to identify factors contributing to these predispositions such as environmental factors, racial traumas, and other socioeconomic factors. Some of these disparities can also be attributed to the effects of the criminal legal system, such as living conditions of prisons and jails and health upon reentry into communities after incarceration [15]. As noted by Bui et al., “[i]ncarcerated persons largely come from disadvantaged communities and return to under-resourced neighborhoods and adverse conditions. In addition to pressing needs such as housing, employment, and education, barriers that negatively affect health can pose substantial challenges for returning citizens” [14], p. 5.

In May 2020, the World Health Organization (WHO) declared the pandemic a global health crisis, escalating from the declaration of a public health emergency of international concern three months prior [16]. This crisis further complicated health disparities faced by CLI individuals as many continued to work as essential personnel throughout the pandemic and remained at higher risk of being exposed to COVID-19 in public settings. Individuals who were incarcerated during COVID-19 were even more likely to be exposed in prisons and jails as these settings facilitated “epicenters of disease” due to poor ventilation, unsanitary conditions, limited access to care, and overcrowding [10], p. 480. In addition to socioeconomic conditions that increase CLI individuals’ risk of COVID-19, prevention and protection measures recommended by the CDC changed rapidly as researchers learned more information about the novel virus, further impacting how people made sense of the virus and how they decided to take up recommended prevention measures.

In December 2020, the CDC began recommending COVID-19 vaccinations to those 16 years and older as the best means of protection. Additionally, hesitancy toward the COVID-19 vaccines added another barrier to the uptake of CDC recommendations. In 2020, researchers conducted a study within correctional and detention facilities across four states and found that 45.4% of people who participated stated they would refuse a COVID-19 vaccine if it were offered to them and 9.7% shared they would be hesitant to receive it [17]. The same survey also found that people who identified as Black reported higher hesitancy toward the vaccine and identified historical mistreatment and higher rates of distrust of health care, correctional and governmental institutions as influencing factors [17], p. 475. Hispanic people and non-Hispanic Black or African American people were twice as likely to be hospitalized for COVID-19 than individuals who identified as non-Hispanic White, a trend that continued through 2022 [18]. Additionally, pre-existing medical conditions, type of work, physical location, access to health care, and racism are all factors researchers have identified as contributing factors. Thus, it is important to understand how CLI individuals’ experiences may have been complicated by these rapid changes in public health guidance, along with the influx of both accurate and false information about COVID-19 from many sources.

## 2. Misinformation in an ‘Infodemic’

A distinct characteristic of the COVID-19 pandemic is a surge of misinformation and disinformation circulating across media and telecommunication platforms [19,20]. We understand misinformation as a phenomenon where people “confidently hold wrong beliefs” [21], p. 791, which are directly influenced by perceptions of evidence, experts, and environments [22]. While misinformation is not a new phenomenon to public health crises [23], the informational environment surrounding COVID-19 is also characterized by the rampant spread of *disinformation*. Distinguishing the two, Rubin argues that “misinformation is unintentional and includes errors or inaccuracies, while disinformation is deliberately deceptive, false or misleading” [24], p. 1017.

The World Health Organization (WHO) declared the current pandemic an infodemic, defined as “an overabundance of information…includ[ing] deliberate attempts to disseminate wrong information to undermine the public health response and advance alternative groups of individuals” [25], para. 2. With COVID-19 causing global death and illness, misinformation and disinformation present a new, heightened threat that “costs lives” [25], para. 3. The infodemic accelerated the destruction of COVID-19 and hindered efficient solutions, as “an anxious public finds it difficult to distinguish between evidence-based information and a broad range of unreliable misinformation” [26], p. 1. Thus, one objective of this study is to explore how the infodemic shaped the sensemaking processes among those impacted by the criminal legal system and how misinformation impacted health behaviors among CLI individuals.

## 3. Health Disparities and Structural Violence

The disproportionate effects of COVID-19 risk exposure and the impacts of misinformation and disinformation may be understood critically by examining the way structural systems are connected to material resources. Societal structures such as medical services, jobs, transportation, food, shelter, and other systems are dependent on individual access to material resources [27]. In relation to COVID-19 and marginalized populations, access to protective measures such as testing and vaccinations, trust in healthcare and governmental organizations, and access to accurate information are important to consider in exploring disparities and their role in the sensemaking process. It is important to note that marginalized populations are disproportionately affected by comorbidities such as obesity or diabetes, which can increase the risk of serious outcomes for contracting COVID-19 [2].

An indirect harm that marginalized populations, including those with criminal legal involvement, face as a result of socioeconomic barriers is structural violence. According to Farmer et al., the roots of structural violence “are embedded in the political and economic organization of our social world; they are violent because they cause injury to people (typically, not those responsible for perpetuating such inequalities)” [28], p. 1686. Working within this understanding, Ramaswamy et al. studied how CLI women navigate the pandemic, chronic illness, homelessness, and shelter-in-place orders [11]. The authors determined that despite elevated risk and prevention challenges, these women “fall outside of targeted efforts to reach people held in jails and prisons” [11], p. 544. Stressful and unstable housing situations were a common theme among participants, directly impacting their ability to comply with public health behaviors such as social distancing during the COVID-19 pandemic.

Structural violence is also situated within the historical context of harm perpetrated by medical authorities against vulnerable communities [29,30,31]. Medical mistrust and institutional distrust among Black communities may be traced to legacies of mistreatment and human rights violations in the history of public health [19,32,33,34,35]. Furthermore, the recent killings of unarmed Black people, such as Breonna Taylor and George Floyd, amplified the harm in legal systems of policing and incarceration that disproportionately impact Black communities [4,36,37]. From the perspective of public health messaging, a lack of trust in authority figures and the government is a critical barrier for this CLI population.

## 4. Making Sense of Uncertainty: Theoretical Framework

As the pandemic evolves and remains unpredictable, uncertainty is a collective global experience. We define uncertainty as not having enough information about a situation to formulate an expected outcome [38]. The desire to control outcomes has been normalized [39], so uncertainty motivates people to seek new information and make sense of the unknown. As individuals engage in “sensemaking”, or ascribing meaning to an event [40], access to accurate information may reduce uncertainty whereas misinformation may increase fear, anger, and uncertainty [41]. Weick’s model of sensemaking is the theoretical framework for this study, which informs the examination of CJI individuals’ experiences in relation to COVID-19 uncertainty [42].

Central to Weick’s Model is the concept of information equivocality, which asserts that increased information about a health issue may reduce ambiguity and uncertainty. Some health issues are more equivocal in nature, and the magnitude and global devastation of COVID-19 present unparalleled levels of uncertainty. Consequently, with less equivocality or uncertainty, individuals are able to manage a health threat with clear, effective coping strategies and achieve better outcomes. In health communication scholarship, the model explores the organization of healthcare services, differing levels of care and relationships within these systems, and how patients make sense of diagnoses [42,43,44,45,46].

Understanding how people navigate information and decision making around COVID-19 requires acknowledging uncertainty as the backdrop of the sensemaking process. As Maitlis and Sonenshein indicate, “Sensemaking is thus about connecting cues and frames to create an account of what is going on” [47], p. 552. In a context of escalating structural injustices, identifying how these cues and frames are formed will allow us to understand how people make sense of COVID-19 information, and illuminate how this process impacts health behavior. Sensemaking has been applied to understand trauma, loss, and identity development for those impacted by the criminal legal system [48], and has been argued as a “common and essential task” in information processing. This process is inherently influenced by intersecting elements of a person’s identity, along with the structures an individual interacts with based on those identities. “The concept of intersectionality describes the ways in which systems of inequality based on gender, race, ethnicity, sexual orientation, gender identity, disability, class and other forms of discrimination; intersect’ to create unique dynamics and effects” [49], para. 1. Understanding how CLI individuals’ intersecting identities influence their sensemaking process regarding misinformation, including disinformation, during COVID-19 is important for contextualizing their health decisions and choice-making processes.

The current study provides a qualitative exploration of the experiences of people who have been involved with the criminal legal system at any point within the previous five years to understand COVID-19 effects on their lives ‘outside’, with a specific interest in how they navigate and protect themselves amid continuously evolving information about the pandemic. Already more vulnerable to the intersections of systemic inequalities, CLI individuals experience unique risks around misinformation. This study explores the lived experiences of CLI communities as they make sense of information and misinformation surrounding COVID-19. The present study extends scholarship about misinformation, trust, and mistrust and how communities who experience socioeconomic vulnerabilities interpret and make sense of a global health crisis.

## 5. Research Questions

Highlighting the meanings CLI individuals make about the influx of competing information as well as how sensemaking is practiced in their health decision-making, this study poses the following research questions:

RQ1: How do CLI individuals make sense of misinformation/disinformation in the context of COVID-19?

RQ2: What are the narratives of trust/mistrust in the context of COVID-19 among CJI individuals?

RQ3: How do socioeconomic and structural vulnerabilities intersect in CLI individuals’ experiences during the COVID-19 pandemic?

## 6. Methods

### 6.1. Research Design

The primary objective of this paper is to explore narratives of misinformation, trust, and mistrust during the pandemic among CLI communities in three central states (Illinois, Louisiana, and Arkansas). We collected data through seven online focus groups for an in-depth exploration of the target communities’ experiences and narratives of COVID-19 [50]. We designed a semi-structured protocol to elicit general knowledge about COVID-19, awareness of misinformation, trusted or distrusted sources, attitudes about COVID-19 health behaviors (including testing, protective behaviors such as mask-wearing and social distancing, and vaccination), and experiences with the criminal legal system during the pandemic. The focus group process was iterative and flexible due to evolving guidance on protective behaviors and health protocols from the CDC. Researchers adapted the protocol as needed to capture questions relevant to new developments, including vaccine rollout.

### 6.2. Participants

Participants were recruited from four counties across the three states—West Cook County and South Cook County, Illinois; Baton Rouge, Louisiana; and Pulaski County, Arkansas—based on partnership with hospitals and Federal Qualified Clinics (FQCs) at these geographic locations. These sites were advantageous as relationships between hospital or FQC staff and people with criminal legal histories supported effective recruitment within strict time frames. Following Institutional Review Board approval, the research team conducted seven online, synchronous, mediated focus groups between December 2020 and January 2021. Each group included four to ten participants and lasted approximately 90 min.

Participants were recruited by staff from participating sites and via snowball sampling as we encouraged prior participants to share flyers with their networks. Each of the sites had a community liaison who assisted with referring potential participants and posting flyers in identified locations. Flyers were distributed at reception when individuals entered FQCs, at individual meetings with the community liaisons, in public places such as FQC waiting rooms, and through advertisements sent out via FQC social media accounts. Only those who were interested in participating in the study continued with the screening process. To qualify to participate in the focus group, those who were interested were asked a screener question, “Have you been to jail, prison and/or on probation/parole within the last five years?” If participants said yes, they were sent an email or text message to join the study. Forty-four of those recruited participated in the study.

Nearly half (47.7%) were located in Illinois, with the remaining participants divided between Arkansas (29.5%) and Louisiana (22.7%). A majority of participants identified as men (54.5%), 13 (29.5%) as women, and four as “another gender” (9.1%). Most participants (36) were between 18 and 30 (45.5%) or 31 and 44 (36.4%) years old. Participants self-identified as predominantly Black or African American (40, 90.9%), including five individuals noting Hispanic or Latinx ethnicity. In terms of CLI experience, the sample reported an average of 5.6 arrests (*SD* = 5.4), 4.2 times in jail or prison (*SD* = 5.2), and 2.0 times on probation or parole (*SD* = 1.9). At the time of the focus group, 29 participants (65.9%) had tested for COVID-19 at least once. (See Table 1 for additional demographic information).

### 6.3. Procedure

Due to COVID-19 restrictions mandating physical distancing, focus groups were conducted virtually utilizing HIPAA-compliant Zoom software. Studies have substantiated the effectiveness of virtual focus groups, specifically Zoom video conferencing, as an effective tool for qualitative research [2,51]. After screening participants to verify CLI, age, and availability, the research team sent reminders three days before, one day before, and within the hour of each scheduled focus group. Reminders and meeting information was sent via text, email, or phone based on participants’ preferred communication style.

Upon entering the Zoom meeting, participants were directed to the chat box for the link to a brief survey, administered through Qualtrics. The survey included information about the study, questions about demographics and COVID-19 awareness, and details to receive an incentive. Informed consent was obtained at the beginning of the survey, including consent for audio and video recording. Participants had the option to turn their cameras off, and those who did not wish to be audio recorded had the opportunity to leave. Once all participants completed the 10- to 15-min brief questionnaire, the recording began and researchers obtained verbal consent again. Participants were compensated for their time with USD 50 through CashApp. All focus groups were conducted in the evening after work hours and co-led by two research team members. Each session was open to participants from all four sites.

## 7. Data Analysis

Four research team members engaged in a two-phase collaborative coding process, which involved a combination of inductive and deductive reasoning [52]. Zoom software provided initial transcripts for each focus group, which were reviewed and edited for accuracy and de-identified using numerical identifiers. All transcripts were linked to Atlas.ti (v9) for data management, coding, and analysis. In the first phase, the research team open-coded six transcripts, yielding 229 total codes. Following an abductive process, we developed a code list utilizing open coding as well as scholarship on misinformation, disinformation, trust, and mistrust [50,52].

In the second phase, the research team reduced the 229 codes into 15 higher-level codes based on thematic similarity and frequency through an iterative process. Any coding discrepancies were resolved using dialogue to reach a consensus. Using the Atlas.ti intercoder agreement function, the research team independently coded one focus group transcript (approximately 14% of all focus group data) with the 15 higher-level codes. Results indicated high intercoder reliability overall with Krippendorf’s alpha (α = 0.91) [53]. The codebook was finalized after establishing intercoder reliability. To complete the coding process, the research team then recoded the six remaining transcripts [52]. To ensure the trustworthiness, flexibility, reflexivity, and transparency required for qualitative rigor, weekly meetings were held throughout the coding process [52,54].

Using interpretative narrative analysis, themes were abductively defined based on a thorough examination of the codes about the participants’ experiences during the pandemic [52]. Through axial coding (second-cycle coding based on an interpretive process), the researchers identified patterns and themes that represented participants’ lived experiences [50,52,55]. Further inductive examination revealed a meta-theme that cut across all findings. We used Weick’s sensemaking framework to interpret and present the results of the data analysis [56,57].

## 8. Results

Three themes were identified based on the research questions: (1) *misinformation and disinformation*—where participants discussed what they perceived to be accurate and false information about the pandemic; (2) *trust and distrust*—where participants described both formal and informal sources of information and the degrees to which they trusted and distrusted them; and (3) *community vulnerability*—where participants identified their own ‘community’ and discussed its complex vulnerability to COVID-19 based on CLI, socioeconomic status, and/or structural racism. Each theme appeared across all focus groups and among most participants, and the meta-theme of uncertainty (i.e., how participants were navigating uncertainty regarding misinformation, the struggle of trust with sources, and the lack of understanding of the pandemic’s influence on their future) was reflected throughout all three themes. Our data also suggested an underlying concern over uncertainty management through sensemaking, which will be addressed further in the Section 9 of this paper. In the quotes below, pseudonyms are used to protect the identities of participants.

### 8.1. Theme 1: Uncertainty Fueled by Misinformation and Disinformation

The first theme illustrates participants’ awareness of misinformation and disinformation pertaining to COVID-19, public health messaging about COVID-19, and overall knowledge about the virus. Participants expressed frustration around constant changes in information and shared their range of information sources, from healthcare workers to news outlets and, at times, social media. While participants shared some of the misinformation and controversial messaging surrounding COVID-19, they also intentionally noted that they did not believe some of the more eccentric messaging surrounding the pandemic, and many were quick to denounce some of the ‘conspiracy theories’. Arie shared:

*Some think that if you’re O positive that you can’t get it. Because your blood type has something to do with it. But if you’re O positive then you won’t get it, and I just don’t believe that…I heard it from my boyfriend. Okay. And he works for the state.*

Terry emphasized awareness of misinformation:

*Well, there’s a large portion of African Americans who are O positive. Yeah, that is the most common blood type, O positive, and if that were true, it wouldn’t be that many deaths.*

However, there were also instances where participants showed strong belief in misinformation. The following quote demonstrates misinformation about COVID-19 and the newly developed vaccines:

*It was a simulation done on this...I think it might be population control, but then you know you don’t know what to believe. I do know they couldn’t possibly produce no rapid vaccine like this unless they already had a plan to have it in the making already.*

(Jordan)

The focus groups occurred nine months following the CDC’s declaration of COVID-19 as a global pandemic, many participants were knowledgeable about virus symptoms and ways to reduce spread. However, some participants were incarcerated at the time of declaration and when released lacked critical information about the nature of the pandemic.

*Well, I just recently came home. I was in a halfway house but before I came to the halfway house I was in jail. And the guy he was making masks and he kept asking me, ‘Hey you want a mask man?’ and I asked, ‘What I want a mask for?’ and he was like, ‘Man, the corona.’ And I was like, ‘What’s corona?’ and he said the President said something about corona. So, I didn’t really think nothing of it, then one of the ladies, one of the guards came into work and was like, ‘It’s here.’…When it first came, I thought it was a joke, but when people start dying, I was like this shit serious.*

(Thomas)

In the face of limited knowledge, participants expressed uncertainty about information sources and about changing guidelines. The struggle to sort through inconsistent information is highlighted in the following quote by Earl who attended a late-January focus group:

*First, it went from not wearing no mask to one mask to now they saying you have to wear two masks. …and I hear a lot of it [misinformation] coming from government-based officials and government-based medical personnel.*

In addition to uncertainty about inconsistencies or changes in health information, participants also shared uncertainty about the efficacy of the protective behaviors recommended by the CDC. Participants were knowledgeable about recommended protective behaviors but were not fully convinced that those behaviors would reduce exposure to COVID-19. While many participants felt that protective behaviors such as mask-wearing and social distancing were acceptable, there was greater hesitancy about the vaccines due to mis/disinformation. For example, Felton said:

*I think I’ll just take my chances on how I’ve been doing and stay social distance and keep that mask on and just try to stay...keep everything sanitized. I don’t have a bad immune system and my kids don’t either…we really been pretty much straight. I’m just not taking that vaccine. No, I’m not doing that!*

Participants worried about the feasibility of protective behaviors amid inconsistent implementation of these behaviors as a precaution. This was heightened for participants who were incarcerated at any point during the pandemic, as Jeremy noted:

*Everybody is not following the guidelines. They not wearing a mask and, you know, they want us to wear a mask but for some reason, I don’t know why they feel like they don’t gotta or like they won’t get it. I just don’t understand that. Where I was- I was locked up at, they want us to do it and stay six feet apart but they putting us right beside each other. So, I don’t understand that. Then, every day and every hour someone new is coming to jail. So... they not taking the proper precautions that I feel to stop the COVID spread.*

### 8.2. Theme 2: Uncertainty Based on Trust Issues with the U.S. Government

The second theme highlights the uncertainty participants experienced as they navigated trust and distrust about COVID-19 overall, vaccines, and information sources. A lack of faith in the government, rooted in historical mistreatment and present systemic barriers, was evident. Suggestions and recommendations for COVID-19 safety changed regularly, further fueling mistrust in the government and other authorities such as the CDC. As a result, participants expressed mixed feelings about the motivations of these sources. Derrick shared the following comment about immunologist, Dr. Kizzmekia Corbett, a Black woman researcher who made important contributions to the development of the Moderna vaccine (Garnett, 2020):

*I just seen they [CDC] put something out where it’s an African American Black lady helping find a cure and it feels like to me y’all are pretty much, I don’t want to say they think we’re stupid, but they trying to coerce us and pander to us, “Oh, we got a Black lady.” And it’s just really funny how the virus is affecting the African American, Latino, and minorities even more, and they’re saying the preexisting conditions, but, you know, at the same time…it’s just one of those things where it kind of seems like it was calculated.*

Clearly, uncertainty about the motivations of the government both arose from and exacerbated the perceptions of mistrust or distrust. Some participants shared that choosing African American spokespeople to encourage vaccination did not help to reduce fears based on historical reasons for mistrust. The deep connection between perceived vulnerability and historical mistreatment and systemic barriers was evident in the following quote:

*Let me say this again. That’s why a lot of us [Black people] don’t trust the government…this goes along with our other stuff, the Tuskegee experiment and all this other stuff that’s been happening to people like that, then you gotta do your own research and things like that, because them son of a guns is slick and trifling…The police shooting all these people and all this stuff, then covering it up and ain’t getting punished for it. So come on now.*

(Dante)

The above quote illustrates the ways in which historical and political contexts influenced participants’ current experiences. Moreover, participants discussed the lack of trust in the political climate, including media reporting on the pandemic.

*“And I hate to say it like this, but from the President [Trump] really...the truth...You know, people don’t believe nothing he say...so everyone’s like, ehhhh, I don’t know. He was in office when it came through. I don’t know. It’s kinda iffy. Nobody trusts the news like the way they used to. We used to go to the news or read the newspaper for real news...now you don’t even believe it. You think it’s a conspiracy and all that.”*

(Toni)

As more information about COVID-19 vaccines became available, participants found themselves reasoning through this information and trying to identify underlying motivations. They were often skeptical and tried to make sense of the urgency of vaccine development based on their perceptions about the government’s response to other public health issues that has negatively impacted their community, such as sickle cell anemia. Timothy shared:

*Yeah, I think it’s about money, you know, the government is in, you know, the health industry is about money and it’s like, y’all don’t have a cure, but y’all got a vaccine this quick when we got like other diseases. Sickle cell or other things that we have not, you know, been able to find any type of help with and all the sudden COVID comes out and then within months there’s a vaccine so it’s just really, really fishy to me.*

While trust in government and medical authorities was relatively low, participants expressed high trust in some alternative sources. Derrick referred to a controversial but well-known herbalist in the Black community, Dr. Sebi (Collins-Dexter, 2020), as a reason to endorse homeopathic alternatives to the vaccine:

*As melanated Black people, one thing that Dr. Sebi taught us is that a virus or sickness cannot live in an alkaline body...if you keep your body in an alkaline state, you’d be very difficult to catch and be susceptible to stuff so one quick tip, you can make your own alkaline water. Get you some lime and cucumber, cut it up in there, drink that. And, you know, drink a lot of teas at least, but research that and we can all get through this.*

### 8.3. Theme 3: Community Vulnerability

The last theme focuses specifically on participants’ vulnerability in relation to intersecting identities or social categories (e.g., CLI status, substance use disorder and recovery, race/ethnicity, living conditions, prior health conditions, etc.). For most, their vulnerabilities were centered around racism and other forms of socioeconomic oppression. Not having adequate income due to COVID-19 disruption, for example, influenced participants’ attitudes toward protective measures and distrust in the government. Even though participants may have been fearful and indicated mistrust of government institutions, they also recognized that this is a worldwide life and death event and therefore they were inclined to follow the recommended procedures such as testing, to continue to protect themselves and others. For example, Sharrell shared their concern for their parent:

*It’s more scary than anything for me because if I contracted it and give it to my mom or something like that. Like, that’s where my fear is right now…Yeah, the six feet thing, washing hands, spraying Lysol, you know, I’ve heard everything from that to just staying inside. So, um, my biggest thing is just learning more about what we can do to prevent it and staying safe.*

Jonathan believed that COVID-19 was intentionally placed within marginalized communities, sharing their inability to access healthcare due to income barriers:

*I think they make sure they put it [the virus] in the smaller minority areas where people don’t have that much healthcare. You don’t go to the hospital because they don’t have the money to go to the hospital…You’ll just drop off and they’ll be like, you know, “Okay, it’s less people we have to worry about now. Okay we’re gonna put it out there with a couple of celebrities so they get it or whatever,” but they okay because they got the money to pay for the healthcare but on the other note everybody else. Y’all just have to wing it.*

Social and economic disruption due to COVID-19 were most acutely felt by those who did not have the option to work from their homes or to not work at all. As a result, participants were left to figure out a way to continue their lives amid the disruption in order to survive. Participants pointed to economic challenges including a lack of housing or phone access as barriers to getting the correct information.

Brandi described the pressures of losing her job, caring for her children, and the reality of losing people to COVID-19:

*Prior to COVID I was doing okay. I ended up getting laid off…I was sitting with a client that was bed bound and I was basically like his direct service worker…she [the employer] didn’t have the money to pay us. I just started driving for Amazon...and it’s like, I’m finally kind of getting back on top of things, like bills and all of that stuff is getting behind. I have two kids…So, it’s like everything is on me. I was like, really stressed out behind it. I personally knew a few people that died from COVID. From the jump, I believed it was real.*

A majority of participants did not identify CLI as a key aspect of their social identity that made them more susceptible to COVID-19. However, the exception would be participants who were incarcerated or in transitional housing during the pandemic. In those cases, participants emphasized increased vulnerability to contracting COVID-19. Gary recalled:

*The county [jail] is already overcrowded to capacity and they’ve had to release a lot of people...but at the same time, they really don’t give a damn...They treat you like animals and dogs anyway, so I’m sure they’re not being proactive about it...I don’t want to say that they’re using this to kill us, but they could care less. They’re like, “Oh, well that’s just another criminal that I don’t have to worry about.”*

Experiences of disruption, incarceration, death, and trauma related to COVID-19 influenced the ways participants understood their vulnerabilities. The difficulties of navigating the pandemic within participants’ life environments were highlighted in their stories about family, community, and the impacts that COVID-19 might have long-term. While making sense of uncertainty is not an easy task, many participants found motivation through spirituality, often referring to a higher power helping them to make it through these difficulties. Previous experiences with substance use disorder or CLI status also influenced attitudes toward resilience.

*I’m actually a recovering addict and if I didn’t let the drugs and alcohol kill me, then I’m not gonna let a pandemic kill me, you know? And God’s got my back. So I stay prayed up and I do what I have to do every day, you know, and I live day by day in recovery and the addiction is way worse than a pandemic and I take the precautions that they tell me to take, exactly like the precautions that I’ve taken in my recovery. So I stay prayed up and I do what I have to do every day.*

(Elias)

Additionally, the focus group itself served as a meaningful mechanism for shared sensemaking. Sherri’s statement sums up common sentiments of how sharing narratives could lead to potential behavioral change:

*I want to thank you guys too because it was a great opportunity…I learned a lot of things. I might just go take a test tomorrow...It’s the words of encouragement. Like the lady told me she had a family member who had diabetes and came out like a champ. And he had it, his mother had it and everything was cool. I mean just the faith, of God I guess. So I don’t know, maybe I might go get a test soon. Y’all just pray for me!*

## 9. Discussion

This study explores the lived experiences and narratives about COVID-19 among individuals with CLI. Results clustered around three themes: (1) *misinformation and disinformation*, (2) *trust and distrust*, and (3) *community vulnerability*. The first theme of misinformation and disinformation reflects the a priori objective of this study, based on prior scholarship surrounding pandemics and misinformation. Weick’s sensemaking theory proposes three stages for organizing to reduce equivocality in health contexts: (1) *the enactment phase (i.e., sensemaking)*, the process where individuals begin to understand the health challenge and to assign meaning; (2) *the selection phase*, the process of selecting which course of action to take in response to the health challenge; and (c) *the retention phase*, the process of reflecting on the enactment and selection phases to guide future behavior [58]. Our data suggest that the concept of equivocality was at the core of the narratives shared among participants, with *uncertainty* emerging as a meta-theme representing how all three themes are interlinked. Our results reveal that participants engaged in sensemaking processes based on experiences of belonging to disenfranchised and minoritized communities.

Although the study targeted those with recent criminal legal involvement, the majority of our sample was Black participants, which speaks to the intersecting challenges exacerbated by the pandemic based on race, socioeconomic status, and CLI impact. Consequently, most of the participants indicated that belonging to a racially minoritized group impacted their perceived risk of infection with COVID-19 and their mistrust of the vaccine. Throughout the focus groups, participants alluded to having been through difficult times before based on intersecting experiences of the legal system, socioeconomic inequity, and racial discrimination. As a result, they were able to hold a perspective of resilience and perseverance by pushing through in the midst of the uncertainty about the pandemic.

### 9.1. Theoretical and Practical Implications

The results of this study clearly outline the process of reducing uncertainty during the pandemic among those involved with the criminal legal system. Weick’s model identifies three stages in the uncertainty management process, namely enactment, selection, and retention; and all three stages were illustrated in the focus group findings. The focus group discussions often began with the enactment and bracketing of various types of information about COVID-19. From the initial alerts of COVID-19 as a global pandemic, there was mystery and confusion surrounding the source and transmission of the virus, ways in which infection could occur, and preventative measures that should be taken. Participants had an acute awareness of misinformation, disinformation, and even conspiracy theories that were spread throughout their communities and social networks. These forms of misinformation connect to recent studies about the fears of historical racist exploitation within the scientific community and healthcare system [33,57]. While reluctant to identify any misinformation that they personally endorsed, participants showed interest in discussing the ideas of population control of Black people and tracking devices through testing and vaccination.

We also observed the presence of an “infodemic” created an environment that exacerbated equivocality, leading to a heightened sense of mistrust and greater barriers in the selection phase of organizing information. Participants indicated an overload in trying to select which information to trust to make an informed decision about the health of themselves, their loved ones, and their communities. The misinformation theme is compounded by the lack of trust that members of criminal legal impacted communities have in scientific and government institutions. During these discussions of mistrust and distrust, fears of anticipated negative effects of COVID-19, testing, vaccinations, and long-term impacts were also expressed. As the pandemic progressed, uncertainty also evolved and shifted. For example, as more information about COVID-19 transmission became available, participants became increasingly knowledgeable about transmission and made sense of the fear they had because of that knowledge. As mandates changed and vaccines were developed, participants found themselves making sense of those shifting uncertainties and associated fears through the integration of new knowledge and reinterpretation of past and current experiences.

Interestingly, participants engaged in the selection phase not only relied on their own knowledge and trusted information sources, including local healthcare providers; but they also used focus groups as an opportunity to enhance sensemaking, actively engage in conversation, and share their own experiences. Participants were selective in which information they trusted, based on personal experiences with sources, and frequently inquired about the moderators’ beliefs and trusted sources for accurate information. However, experience with the criminal legal system intensified mistrust among participants that belonged to a racial minoritized community. Therefore, the impact of the criminal legal system intersected with participants’ mistrust and reinforced the fears behind believing messages even from “reputable” sources, such as the CDC and Dr. Kizzmekia Corbett. Thus, this study provides nuance to the conceptualization of trust and distrust among Black and CLI communities toward public health organizations.

Furthermore, this study provides insight into the retention phase of sensemaking, which is described as deciding on the action to take next or health behavior [56]. Economic disruption because of COVID-19, and the subsequent global shutdowns to curb the pandemic, were mostly felt by those who had to go to work with no option of working from home. As a result, participants were left to figure out a way to continue their lives amid the disruption for their own survival. Without the ability to work remotely, many participants shared that they were continuing to work outside of the home and often relied on public transportation. In addition to increased exposure, participants often worried about the feasibility and implementation of protective behaviors. Furthermore, participants expressed concern about the personal impact of contracting COVID-19 and the long-term effects of the pandemic on the economy. In essence, participants displayed the challenges with sensemaking due to chronic uncertainty and previous and ongoing structural violence and “syndemic” conditions (i.e., multiple, and co-occurring epidemics) [4,34,36,37].

The alignment of our findings with Weick’s perspective on equivocality reduction suggests that the sensemaking process during the COVID pandemic and practices in the CLI communities during the pandemic are inherently systematic, but also complicated [58]. Our study furthers the theoretical application of Weick’s sensemaking process in an underresearched healthcare context including the interplay of mis/disinformation, mistrust, and other forms of community vulnerability. These insights could inform further theoretical examination and empirical study of sensemaking in the context of health disparities and inequity. Our findings also have practical implications for community operators and policy makers. First, the sources through which health information is passed are a determining factor for each stage of the sensemaking process; thus, health information sources and figureheads must be thoughtfully considered from a sociocultural and historical standpoint. For example, participants were less likely to enact information from sources with past transgressions and that have harmed vulnerable communities. Second, reducing precarity and addressing their socioeconomic needs were the most important concerns for CLI individuals; thus, identifying ways to connect CLI individuals to tangible resources such as housing, food, employment, childcare, etc., is necessary for any attempt toward reducing equivocality in sensemaking. Lastly, combatting assumptions that misinformation only shows in the form of “conspiracy theories” is necessary for better understanding the experiences of CLI individuals. Much of the information participants identified as misinformation stemmed from their lived experiences, historical context, and/or the current political climate. Community operators and policy makers should seek to further understand the perspectives of CLI individuals and the ways in which they communicate about their sensemaking processes as it sheds light on broader societal relations.

### 9.2. Limitations and Future Research

There are a few limitations to note. This study recruited those with CLI experience at any point within the previous five years. Future studies should identify differences in experiences based on timing and length of incarceration in relation to participation in the study. Additionally, while the ability to meet virtually allowed for the study to occur during a time of restricted and limited travel, reliable internet access was not always available. During recruitment, a secure space for participants was offered for some of the FQC and clinic sites but not all. Potential participants without personal internet or Zoom access might have felt discouraged from participating. While we did not segregate focus groups based on location, different policies that were mandated based on state regulations (e.g., mask-wearing) could have introduced heterogeneity in participant experiences and perspectives.

A critical strength of this study is its unique sample, which allowed for the centering and close examination of the experiences of a key marginalized group. The qualitative design of the study allowed for a deep analysis of the intersectional vulnerabilities of the study population, providing novel evidence of the complex and nuanced ways in which misinformation and mistrust could impact perceptions and behaviors among CLI communities. Finally, utilizing the Zoom platform provided an effective and feasible way to gather qualitative data. Participants were fully engaged and viewed the focus group as a source of social support, realizing that they were not alone in managing the uncertainty of the pandemic. We suggest that future studies explore the methodology of focus groups as an intentional space of sensemaking.

Future studies should engage more with this population to understand the intersections of risks and vulnerabilities. Although many protective behaviors were recommended to reduce the spread of COVID-19, marginalized populations were disproportionately affected. As stated by Bowleg, “COVID-19 reveals disproportionate risk and impact based on structured inequality at intersections of racial/ethnic minoritized status and class, as well as occupation” [6], p. 917. As the virus continues to spread, health disparities are amplified and vulnerable communities bear the brunt of the impact. This study provides entry for examining further how intersecting vulnerabilities impact CLI individuals’ experience of uncertainty and the associated sensemaking process. Furthermore, using a critical lens and an analysis of power to examine how systems of oppression influence the sensemaking process will benefit public health promotion through message development for marginalized communities.

## 10. Conclusions

This study explored experiences with mis/disinformation, mistrust, and vulnerability among those who were impacted by the criminal legal system. The unprecedented nature of the COVID-19 pandemic resulted in widespread and persisting uncertainty. The political polarization of the pandemic and viral racial reckoning added to the equivocality of the times. Exploring the process of sensemaking from the perspectives of the multiplicity of vulnerabilities faced by CLI communities can lead to translatable insights for public health interventions. This study demonstrates how those who are considered most vulnerable—CLI and minoritized populations—pursue sensemaking amidst misinformation and mistrust, especially given prior experiences that serve to disengage the population. Results in this study connect to the “strategic trust” that Black communities utilize during a public health crisis [33], indicating that an overgeneralization that Black people are less trusting can reduce the effectiveness of public health efforts. The findings of this study should prove useful for those who are developing messaging to combat mis/disinformation and overcome mis/distrust with the medical system and government institutions among disenfranchised communities.

## Figures and Tables

**Table 1:** Sample demographics (*n* = 44).

Variable	*n*	%
Gender		
Man	24	54.5%
Woman	13	29.5%
Another gender	4	9.1%
Not reported	3	6.8%
Race		
Black or African American (non-Hispanic)	35	79.5%
Black or African American and Hispanic or Latinx/e	5	11.4%
White (non-Hispanic)	2	4.5%
Not reported	2	4.5%
Age (Years)		
18 to 30	20	45.5%
31 to 44	16	36.4%
45 to 55	5	11.4%
>55	2	4.5%
Not reported	1	2.3%
Household Income		
USD 0 to USD 9999	17	38.6%
USD 10,000 to USD 19,999	4	9.1%
USD 20,000 to USD 34,999	9	20.5%
USD 35,000 to USD 49,999	4	9.1%
USD 50,000 to USD 74,999	4	9.1%
USD 75,000 to USD 99,999	1	2.3%
Not reported	5	11.4%
Education		
Some high school	7	15.9%
High school	20	45.5%
Trade school	4	9.1%
Associate’s degree	4	9.1%
Bachelor’s degree	6	13.6%
Master’s degree	1	2.3%
Not reported	2	4.6%
Tested for COVID-19 (Self)		
Yes, once	11	25.0%
Yes, multiple times	18	40.9%
No	12	27.3%
Not reported	3	6.8%
*Tested positive for COVID-19*	*4*	*9.1%*
Tested for COVID-19 (Others in Household)		
Yes	11	25.0%
No	30	68.2%
Not reported	3	6.8%
*Tested positive for COVID-19*	*2*	*4.5%*

## Data Availability

The data presented in this study are available on request from the corresponding author. The data are not publicly available due to privacy issues.
